# Ribonucleotide Reductases from Bifidobacteria Contain Multiple Conserved Indels Distinguishing Them from All Other Organisms: *In Silico* Analysis of the Possible Role of a 43 aa Bifidobacteria-Specific Insert in the Class III RNR Homolog

**DOI:** 10.3389/fmicb.2017.01409

**Published:** 2017-07-31

**Authors:** Seema Alnajar, Bijendra Khadka, Radhey S. Gupta

**Affiliations:** Department of Biochemistry and Biomedical Sciences, McMaster University, Hamilton ON, Canada

**Keywords:** novel features of ribonucleotide reductases, probiotic bacteria, *Bifidobacteriales*, conserved signature inserts and deletions, homology modeling and protein docking studies, extended allosteric site, phylogenetic analysis

## Abstract

Bifidobacteria comprises an important group/order of bacteria whose members have widespread usage in the food and health industry due to their health-promoting activity in the human gastrointestinal tract. However, little is known about the underlying molecular properties that are responsible for the probiotic effects of these bacteria. The enzyme ribonucleotide reductase (RNR) plays a key role in all organisms by reducing nucleoside di- or tri- phosphates into corresponding deoxyribose derivatives required for DNA synthesis, and RNR homologs belonging to classes I and III are present in either most or all *Bifidobacteriales.* Comparative analyses of these RNR homologs have identified several novel sequence features in the forms of conserved signature indels (CSIs) that are exclusively found in bifidobacterial RNRs. Specifically, in the large subunit of the aerobic class Ib RNR, three CSIs have been identified that are uniquely found in the *Bifidobacteriales* homologs. Similarly, the large subunit of the anaerobic class III RNR contains five CSIs that are also distinctive characteristics of bifidobacteria. Phylogenetic analyses indicate that these CSIs were introduced in a common ancestor of the *Bifidobacteriales* and retained by all descendants, likely due to their conferring advantageous functional roles. The identified CSIs in the bifidobacterial RNR homologs provide useful tools for further exploration of the novel functional aspects of these important enzymes that are exclusive to these bacteria. We also report here the results of homology modeling studies, which indicate that most of the bifidobacteria-specific CSIs are located within the surface loops of the RNRs, and of these, a large 43 amino acid insert in the class III RNR homolog forms an extension of the allosteric regulatory site known to be essential for protein function. Preliminary docking studies suggest that this large CSI may be playing a role in enhancing the stability of the RNR dimer complex. The possible significances of the identified CSIs, as well as the distribution of RNR homologs in the *Bifidobacteriales*, are discussed.

## Introduction

The *Bifidobacteriales* constitute an important order of bacteria within the phylum Actinobacteria (Ventura et al., 2007; Zhi et al., 2009; Gao and Gupta, 2012). While some species belonging to this order are pathogenic (Smith et al., 1992; Bradshaw et al., 2006; Alves et al., 2014; Kenyon and Osbak, 2014) many *Bifidobacteriales* species belonging to the genus *Bifidobacterium* are known for their beneficial health-promoting effects in humans and other mammals (Gibson et al., 1995; Leahy et al., 2005; Masco et al., 2005; Ventura et al., 2009; Cronin et al., 2011). These probiotic bifidobacteria form a significant constituent in the microbiota of the human colon, and exert their effects as commensal microorganisms (Biavati et al., 2000; Turroni et al., 2008, 2009; Mills et al., 2011; Milani et al., 2014; Ventura et al., 2014). As a result, these bacteria are frequently exploited by the food industry to create consumable products that increase their relative proportion in the gut (Gibson et al., 1995; Sanders, 1998; Masco et al., 2005; Oberg et al., 2011; Ventura et al., 2014). Bifidobacteria are Gram-positive, anaerobic, saccharolytic organisms with a unique metabolic pathway known as the “bifid shunt” (Palframan et al., 2003; Biavati and Mattarelli, 2006; Milani et al., 2015). While many characteristics are known about this important group of bacteria, the biochemical and molecular properties contributing toward their probiotic effects, and adaptability in their respective environments, remain elusive (Ventura et al., 2009; Turroni et al., 2014).

The present study focuses on the enzyme ribonucleotide reductase (RNR), the sole enzyme capable of reducing nucleoside di- or tri- phosphates (NDPs or NTPs) into deoxyribonucleotides (dNDPs or dNTPs) (Eklund et al., 2001; Nordlund and Reichard, 2006; Torrents, 2014). There are currently three recognized classes of RNRs, named classes I, II, and III, sharing no more than 10% sequence identity across their lengths, which are distributed in different organisms (Logan et al., 1999; Sintchak et al., 2002; Torrents et al., 2002). Class I RNR is further divided into three subclasses viz. Ia, Ib, Ic (Jordan et al., 1996; Jiang et al., 2007; Bollinger et al., 2008). The distributions of these different classes of RNRs within the bacterial domain does not follow any specific pattern that can be correlated with the phylogenies of the bacterial phyla (Torrents et al., 2002; Lundin et al., 2009). However, since the different classes of RNR employ different mechanisms of action and require differing environmental prerequisites to function, we explore their distribution in bifidobacteria in an attempt to identify any unique characteristics that may distinguish them.

Each RNR is capable of reducing all four ribonucleotides into their corresponding deoxyribonucleotides by exhibiting a tightly regulated allosteric substrate specificity site, and employing a convoluted mechanism involving radical chemistry that ultimately results in the removal of a hydrogen from the 3′ carbon of the substrate (Brown and Reichard, 1969; Reichard, 1993, 2010; Eriksson et al., 1997; Eliasson et al., 1999). Some RNRs have an additional overall activity site, made possible by the existence of an ATP cone domain at the N-terminus (Thelander and Reichard, 1979). Class I RNRs use NDPs as their substrate, and are aerobic tetramers consisting of one large (R1) and one small (R2) homodimers (Nordlund and Reichard, 2006). The R2 dimer harbors a dinuclear metallocofactor where the radical is formed and subsequently transferred to the active site located at the R1 subunit. Classes Ia, Ib, and Ic differ in the type of metallocofactor in R2 (manganese and/or iron), as well as the different cofactors required for enzymatic function (Petersson et al., 1980; Nordlund and Reichard, 2006; Jiang et al., 2007; Bollinger et al., 2008; Torrents, 2014). Most bifidobacteria harbor a class Ib RNR (Lundin et al., 2009), whose large and small subunit are encoded by the *nrdE* and *nrdF* genes, respectively, and require NrdH as a reductant and NrdI as a cofactor; in contrast, classes Ia and Ic utilize thioredoxin and/or glutaredoxin as reductants, do not require additional cofactors, and their large and small subunits are encoded by *nrdA* and *nrdB*, respectively (Jordan et al., 1997; Cotruvo and Stubbe, 2008; Roca et al., 2008; Crona et al., 2011). Class II RNRs are not oxygen sensitive, use either NDPs or NTPs as their substrate, and are the only monomeric class of RNR (Tamao and Blakley, 1973; Larsson et al., 2010), however, their structural topology mimics a dimer (Sintchak et al., 2002). No known bifidobacteria harbor a class II homolog, but all bifidobacteria possess a class III RNR. Class III RNRs are encoded by *nrdD* and *nrdG* genes and function under strictly anaerobic conditions, with NTPs as their sole substrates (Garriga et al., 1996; Torrents et al., 2001). They consist of a large R1 subunit (NrdD) that is a dimer in its native state, and works concomitantly with a small activase (NrdG) which generates the radical utilizing a [4Fe-4S] cluster (Eliasson et al., 1992; Sun et al., 1995; Logan et al., 1999). This is unlike class I RNRs, where radical formation by the small subunit is required to induce dimer formation of the large subunit (Ollagnier et al., 1996; Torrents, 2014). Despite the described differences in the properties of the different classes of RNRs, the remarkable structural similarities seen across the three main RNRs strongly suggest a common evolutionary origin of them (Poole et al., 2002; Sintchak et al., 2002; Torrents et al., 2002). In all three RNRs, allosteric regulation involves binding of the dNDP/dNTP products at a 4-helix bundle, involving two helices from each monomeric subunit, at the dimer interface of the enzyme (Uhlin and Eklund, 1994; Larsson et al., 2001). The allosteric regulation causes conformational changes at a highly conserved 10 stranded α/β barrel where the active site “finger loop” structure resides in its center, or is brought to its center upon activation (Aurelius et al., 2015).

Although previous studies have significantly contributed to the current understanding of the structure and function of the different RNRs, in the present work we focus on the specific biochemical/molecular properties of the RNRs from bifidobacteria that may shed light on their unique physiological effects. Our earlier work describes a number of conserved signature indels (CSIs) in the homologs of many important proteins from that are uniquely found in all *Bifidobacteriales* (Zhang et al., 2016). These CSIs represent vertically transferred genetic changes that are indicated to have occurred in a common ancestor of the group in which they are found, thus asserting their value as highly specific molecular markers. In the present work, we have performed similar comparative genomic studies that have led to the identification of several novel CSIs in class Ib and III RNR homologs that are shared by all genome-sequenced *Bifidobacteriales* species that contain the respective protein homolog(s), but are absent in all other bacteria. We also describe the results of protein modeling which illustrate the structural location of these CSIs, as well as the results of preliminary *in silico* docking studies which suggest that one of the large CSIs [a 43 amino acid (aa) insertion in class III RNR] may be playing a role in NrdD complex stability.

## Materials and Methods

### Identification of Conserved Signature Indels

The approach used to identify CSIs in RNR was as described in earlier work (Gupta, 2014; Zhang et al., 2016). Multiple sequence alignments (MSAs) were initially created using the Clustal_X 2.1 (Larkin et al., 2007; Goujon et al., 2010) program for the protein sequences of NrdE, NrdF, NrdH, NrdD, and NrdG homologs from about 10–15 *Bifidobacteriales* species, as well as 8–10 species from other groups/phyla of bacteria. These sequence alignments were examined for the presence of conserved indels that are limited to the *Bifidobacteriales* homologs and are flanked on both sides by at least five conserved residues in the neighboring 30–40 aa. A detailed Blastp search (Altschul et al., 1997) was then conducted on the sequence region containing the potential conserved indels to investigate the species-specificities of the identified indels. The indels that were not flanked by conserved regions were not further investigated in our work. The signature files shown here were created using SIG_CREATE and SIG_STYLE from the GLEANS.net program as described in earlier work (Gupta, 2014; Zhang et al., 2016). Unless otherwise indicated, all of the reported CSIs are specific for the *Bifidobacteriales* homologs and similar CSIs were not observed in homologs from any other bacterial species within the top 500–1000 blast hits examined.

### Phylogenetic Tree Construction

In this study we have constructed three separate phylogenetic trees: (i) based on NrdE (large subunit of class Ib RNR) sequences, (ii) based on NrdD (large subunit of class III RNR) sequences, and (iii) based on the large subunit sequences from class I (NrdA, NrdE), II (NrdJ), and III (NrdD) RNRs. For these studies, NrdE and NrdD homologs from all genome sequenced bifidobacterial species were obtained from the NCBI GenBank sequence database (Benson et al., 2017). The species represented in the tree based on NrdD sequences included 49 of 58 validly published *Bifidobacterium* species, the two known *Scardovia* species, all three *Alloscardovia* species, and the single species known from the *Parascardovia* and *Gardnerella* genera. The tree based on NrdE sequences similarly included the subset of these *Bifidobacteriales* species where the protein homolog was detected. Sequences from members of the *Bifidobacteriales* genera *Aeriscardovia* and *Pseudoscardovia* were not available at the present time and were not included in our study. For each tree, a MSA of RNR homologs was created using the Clustal_X 2.1 (Larkin et al., 2007; Goujon et al., 2010) program. For each of these trees, we have additionally included a number of outgroup species (20 species for the NrdD tree, 23 species for the NrdE tree) from other orders in the Actinobacteria phylum, as well as Firmicutes species. For the tree concerning the sequences from large subunits of all RNR classes, we have included <10 NrdE and <10 NrdD sequences from representative *Bifidobacteriales*, in addition to several species across various bacterial phyla in order to depict the evolutionary history of RNR classes. The MEGA 6 program (Tamura et al., 2013) was used to construct a maximum likelihood (ML) tree based on 1000 bootstrap replicates for each alignment employing the Whelan and Goldman model substitution method (Whelan and Goldman, 2001). Gaps and regions with missing data from the sequence alignments were completely removed. In each case, a discrete Gamma distribution was used to model evolutionary rate differences among sites (five categories) and the Jones–Taylor–Thornton substitution method was used to compute the initial trees for the heuristic search using the Neighbor-joining method with a matrix of pairwise distances (Jones et al., 1992).

### Homology Modeling of RNR Homologs and Structural Analysis of CSIs

The approach used to model the CSIs involves homology modeling based on previously crystallized class Ib and III RNR proteins. A Position-Specific-Iterated Blastp search (Altschul et al., 1997) was performed on *Bifidobacterium longum* NrdE (Accession no. EPE39971) and NrdD (Accession no. KXS29127) sequences against the PDB database which revealed that the class Ib RNR from *Salmonella typhimurium* (PDB ID: 1PEQ) (Uppsten et al., 2003) and class III RNR from Enterobacteria phage T4 (PDB ID: 1H7B) (Larsson et al., 2001) exhibited the highest sequence similarity to the *Bifidobacteriales* homologs and provided suitable templates for homology modeling of the RNR isoforms of *B. longum.* A conserved domain search (CD-Search) (Marchler-Bauer and Bryant, 2004) was conducted on the *B. longum* sequences. Homology modeling was performed using MODELLER v9.11 (Eswar et al., 2007) and the top 500 models were initially created and ranked on the basis of their Discrete Optimized Protein Energy (DOPE) scores (Shen and Sali, 2006). The selected models of RNR homologs with the highest DOPE score were then submitted to the GalaxyRefine server (Heo et al., 2013; Lee et al., 2016) to obtain atomic-level energy minimization and to improve the stereochemical quality of the model. The secondary structure elements in the regions containing CSIs were examined and compared with results of the PSIPRED and CONCORD analyses to ensure their reliability (Jones, 1999; Wei et al., 2011; Buchan et al., 2013). The stereochemical properties of the final models were assessed using four independent servers: RAMPAGE, ERRAT, PROSA and Verify3D (Bowie et al., 1991; Luthy et al., 1992; Colovos and Yeates, 1993; Sippl et al., 1999; Lovell et al., 2003; Wiederstein and Sippl, 2007). These validation tools utilize a dataset of highly refined solved structures to evaluate the statistical significance of models based on the conformation, location, and the environment of individual amino acids in the protein sequence, as well as the model’s overall structural stability. The structural alignments of the models with the respective templates were carried out using PyMOL Version 1.8 (Schrödinger, 2016) in order to analyze the location and the structural features of the CSIs in the protein structure. This procedure was followed to create the homology models of both of the RNR homologs found in bifidobacteria. In addition, a structural model of class III RNR was also generated using I-TASSER, an online server that uses threading to predict three dimensional protein structure (Zhang, 2008; Roy et al., 2010; Yang et al., 2015).

### Protein–Protein Docking Analysis of the Class III RNR Homologs

Protein–protein docking studies were performed in order to assess the possible role of a large CSI in the formation or stabilization of the dimeric structure of class III RNR in bifidobacteria. A structural model of the class III RNR from *B. longum* was created by removing the CSI residues from its primary sequence, using the methods described above for other RNR homologs. An additional structural model of RNR was generated with the CSI region constructed as an extended helix. In this structural model, the CSI has a slightly different secondary structure than those of the models that followed PSIPRED/CONCORD, or I-TASSER predictions. This was done in an attempt to be inclusive of multiple possible structural conformations of the CSI. Four structures of the anaerobic RNR monomer viz. PSIPRED/CONCORD based model, I-TASSER generated model, model with extended helix, and the CSI-lacking model, were submitted to two independent web-based protein–protein docking programs using default parameters, viz. PatchDock Version B 1.3 (Schneidman-Duhovny et al., 2005) and ClusPro Version 2.0 (Comeau et al., 2004). PatchDock is an efficient molecular docking algorithm that employs a geometry-based shape complementarity approach which aims to yield refined atomic contacts of protein–protein complexes. Its scoring function takes into consideration both geometric fit and atomic desolvation energy (Duhovny et al., 2002). On the other hand, ClusPro utilizes PIPER, a rigid body docking program, which is based on a novel Fast-Fourier Transform (FTT) docking approach with pairwise potential. Its scoring function is thus based on pairwise interaction potentials (Comeau et al., 2004; Kozakov et al., 2006). The resulting top scoring dimer complex models of RNR from each server (if any) were then refined using the RosettaDock (ROSIE) server (Lyskov and Gray, 2008; Chaudhury et al., 2011; Lyskov et al., 2013). For the docking scores of ClusPro and RosettaDock, the lower (negative) binding energy value indicates improved stability of the docking complexes. In the case of PatchDock, the geometry shape complementarity score was utilized to determine rank, and higher (positive) scores indicate stronger binding affinity (see also notes in **Table 1**). In addition, the monomeric forms of each of the four models were structurally aligned with the established biological assembly of the RNR dimer, and the resulting dimer orientations were utilized as additional inputs for submission to the ROSIE server. The resulting refined structure from ROSIE with the lowest total score, maximum cluster size and the smallest RMSD with respect to the solved structure of RNR complex, was chosen as a representative structure for detail interface interaction analysis. To analyze the dimer interface, this class III RNR dimeric output structure was submitted to the PDBePISA Version 1.48 server, using default parameters (Krissinel and Henrick, 2007).

**Table 1:** Results of protein–protein docking studies for the *Bifidobacterium longum* RNR (NrdD subunit) either containing or lacking the large 43 aa CSI.

Docking server	Protein models of the NrdD		
		CSI-containing	
	CSI-containing	(based on	CSI
	(Extended helix)	PSIPRED/CONCORD)	lacking
ClusPro	-1297.200	-1190.900	-953.000
ClusPro + ROSIE	-1092.851	-1110.039	-1042.091
PatchDock	^∗^	16192.000	11234.000
PatchDock + ROSIE	^∗^	-1109.473	-1033.714
“ROSIE”	-1105.550	-1114.689	-1043.235

*The results of docking studies are shown for two different models of the CSI-containing protein (Extended helix and based on PSIPRED/CONCORD) and compared with those for the model for CSI-lacking protein. The monomers based on the indicated models were submitted to ClusPro and PatchDock servers. Results from the ClusPro and PatchDock servers were also submitted to the ROSIE (RosettaDock) server for local refinement. The results shown under the heading “ROSIE” were obtained by submitting model monomers that were superimposed onto the crystallized 1H7B (Larsson et al., 2001) template dimer. For the ClusPro and ROSIE servers, the lower binding energy (more negative values) is indicative of the increase in stability of the docking complexes, while for the PatchDock server higher (positive) scores indicate stronger binding affinity (see Materials and Methods section for further details). Asterisks (^∗^) indicate that no biologically relevant complexes were obtained from the indicated docking servers.*

## Results

### Identification of Conserved Signature Indels in Class I and Class III RNR Homologs and Their Phylogenetic Implications

**FIGURE 1:**
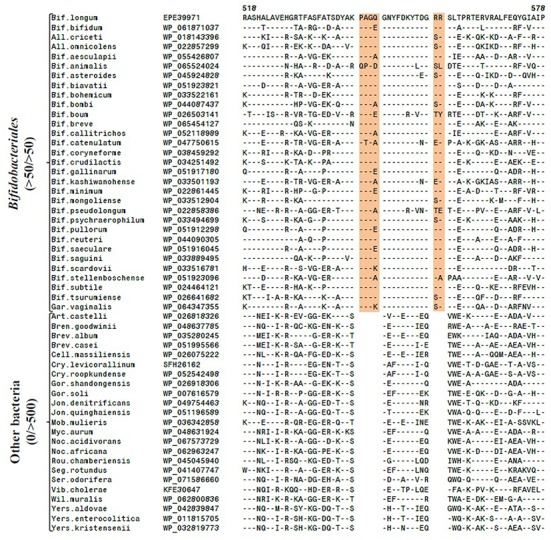
Partial sequence alignment of the large subunit of the class Ib ribonucleotide reductase (NrdE) protein showing two conserved inserts (highlighted) that are exclusively found in all *Bifidobacteriales* members that carry the homolog, but absent in other bacteria. The dashes in the sequence alignment denote identity with the amino acid found on the top line. The Genbank accession numbers of the sequences are shown in the second column. The results are shown for only a limited number of species, however, other species not shown showed similar pattern as described here. Abbreviations used for the genus names are: *All., Alloscardovia; Art., Arthrobacter; Bif., Bifidobacterium; Bren., Brenneria; Brev., Brevibacterium; Cell., Cellulomonas; Cry., Cryobacterium; Gar., Gardnerella; Gor., Gordonia; Jon., Jonesia; Mob., Mobiluncus; Myc., Mycobacterium; Noc., Nocardia; Rou., Rouxiella; Seg., Segniliparus; Ser., Serratia; Vib., Vibrio; Wil., Williamsia; Yer., Yersinia.*

Comparative analysis of the *Bifidobacteriales* genomes indicated that all of the sequenced species from this order contain an anaerobic class III RNR homolog. In addition, an aerobic class I RNR belonging to the class Ib group was also found in most species from this order except *B. adolescentis, B. angulatum, B. dentium*, *B. gallicum, B. cuniculi, B. lemurum, B. merycicum, B. moukalabense, B. ruminantium*, and members of the genera *Parascardovia* and *Scardovia*. The sequences of class Ib and III RNRs were examined for the presence of any CSIs that are specific for bifidobacteria. The results of these studies have identified three CSIs in the large subunit of the class Ib RNR (NrdE) homologs, which are specifically found in the bifidobacterial enzyme. Sequence information for two of these CSIs, which are comprised of 4 and 2 aa inserts in a conserved region of the NrdE protein, is shown in **Figure 1**. As seen in the figure, both of these CSIs are flanked on either side by conserved regions and while they are commonly shared by all of the bifidobacteria harboring the NrdE homolog, they are not present in any other bacterial species in the top 500 blast hits. Sequence information for one additional CSI in the NrdE protein, consisting of a 1 aa deletion also specific for all *Bifidobacteriales* strains that harbor the protein, is presented in Supplementary Figure 1, and is once again absent in other bacteria.

**FIGURE 2:**
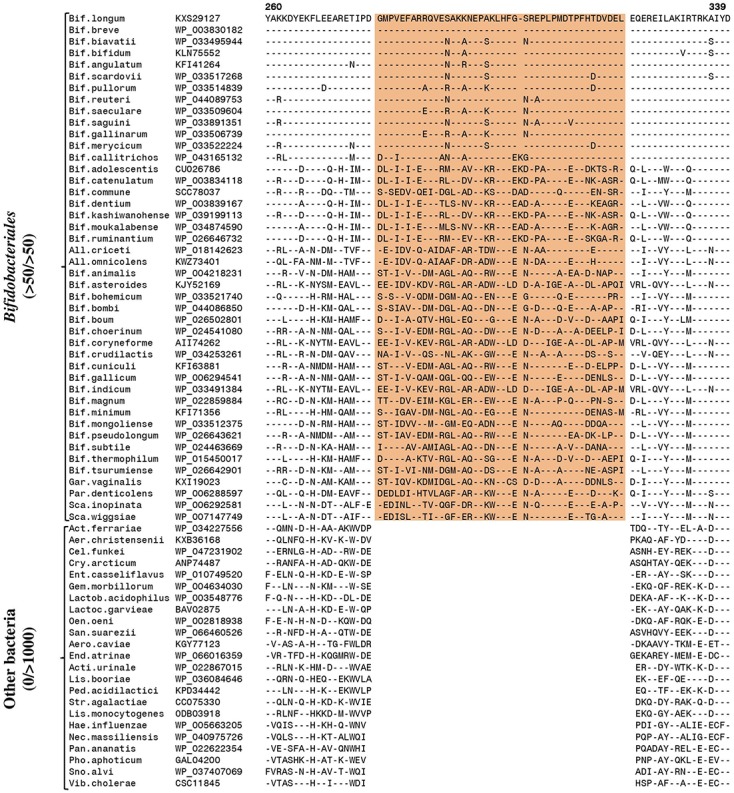
Partial sequence alignment of the large subunit of the class III ribonucleotide reductase (NrdD) protein showing a 43 or 44 amino acid insertion (highlighted) that is exclusively found in all *Bifidobacteriales* members, and absent in other bacteria. Other details are as in **Figure 1**. Abbreviations used for the genus names are: *Act., Actinotalea; Acti., Actinotignum; Aer., Aerococcus; Aero., Aeromonas; All., Alloscardovia; Bif., Bifidobacterium; Cel., Cellulosimicrobium; Cry., Cryobacterium; End., Endozoicomonas; Ent., Enterococcus; Gar., Gardnerella; Gem., Gemella; Hae., Haemophilus; Lactob., Lactobacillus; Lactoc., Lactococcus; Lis., Listeria; Nec., Necropsobacter; Oen., Oenococcus; Pan., Pantoea; Par., Parascardovia; Ped., Pediococcus; Pho., Photobacterium; San., Sanguibacter; Sca., Scardovia; Sno., Snodgrassella; Str., Streptococcus; Vib., Vibrio.*

Similarly, analysis of the sequences from the NrdD homolog has also led to the identification of five CSIs that are specific for the *Bifidobacteriales* homologs. Sequence information for one large CSI, a 43 aa insertion, that is specifically found in the NrdD homolog from bifidobacteria, is presented in **Figure 2**. Sequence information for four other CSIs, consisting of a 1 aa deletion, two 1 aa insertions, and a 4 aa insertion, which are also either exclusively or mainly found in the *Bifidobacteriales* NrdD homologs, are presented in Supplementary Figures 2–5. Of these other CSIs, the 1 aa deletion (Supplementary Figure 3) is also present in *Coriobacteriales* species, which are also anaerobic and saccharolytic bacteria. Additionally, one of the CSIs consisting of a 1 aa insert is also shared by *Lactobacillus* species (Supplementary Figure 2), and the other single aa insert is shared by few other species from the Actinobacteria phylum (Supplementary Figure 5). Asides from these cases, the CSIs were not found in the additional 500–1000 bacterial outgroups examined. The locations of the different identified CSIs over the lengths of the NrdE and NrdD proteins and their respective domains are presented in Supplementary Figures 6, 7. All of the CSIs in the NrdE and NrdD subunits are located in the RNR domain of the respective proteins. In contrast to the NrdE and NrdD proteins, no specific CSIs were found in the NrdF, NrdH, or NrdG proteins. Due to the exclusive presence of most of these CSIs in the RNR homologs from bifidobacteria, they provide molecular markers for distinguishing members of the order *Bifidobacteriales* from other bacteria, and they may inform important differences in the molecular/biochemical properties of the RNR homologs from bifidobacteria. It should be mentioned that in addition to the described CSIs, the NrdE and NrdD homologs from bifidobacteria also harbor other genetic changes such as amino acid substitutions that appear specific for them. Although the evolutionary significance of these changes is not clear and was not studied in the present work, it is likely that some of them also play important role in conjunction with the CSIs in the novel functional aspect(s) of the RNRs from bifidobacteria.

**FIGURE 3:**
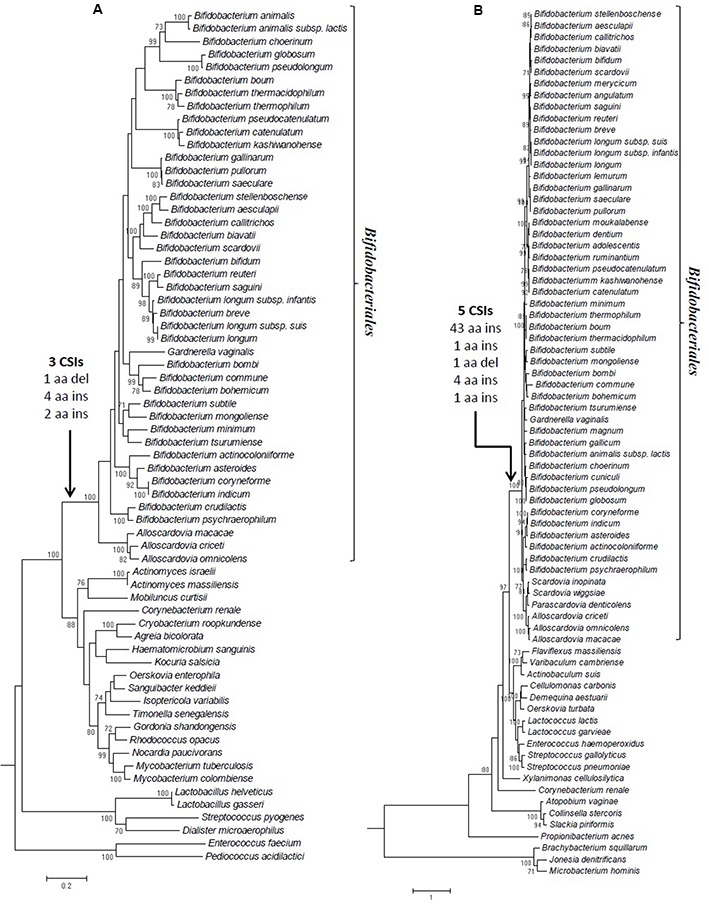
Maximum likelihood trees based on the sequences of **(A)** the large subunit of the class Ib ribonucleotide reductase (NrdE) involving 684 aa residues after the complete deletion of alignment gaps, and **(B)** the large subunit of the class III ribonucleotide reductase (NrdD) involving 549 aa residues after complete deletion. Each tree is based on >40 genome sequenced *Bifidobacteriales* species and ≥20 outgroup members from other Actinobacteria as well as Firmicutes. The trees were constructed using the MEGA6 program and are drawn to scale where horizontal branch length is measured in the inumber of aa substitutions per site. Bootstrap scores that were greater than 70% are shown as a percentage on the nodes. The arrows indicate where the CSI events are likely to have occurred.

Maximum-likelihood phylogenetic trees were constructed for the class Ib and class III RNRs protein sequences based on the NrdE and NrdD proteins, and these trees are shown in **Figures 3A,B**, respectively. In addition to the sequences from a large number of *Bifidobacteriales* species covering the order, the tree also contains information for several other Actinobacteria as well as a limited number of Firmicutes species; the sequences from the Firmicutes species were used to root the trees. The sequences from *Bifidobacteriales* species formed strongly supported monophyletic clades in both trees. Because the sequence alignments used for construction of these phylogenetic trees did not contain any sequence gaps, the observed branching pattern was not influenced by the presence of the identified CSIs. Therefore, the distinct branching of bifidobacteria observed in both trees supports the notion that the reported CSIs in the NrdD and NrdE proteins most likely first occurred in a common ancestor of the order *Bifidobacteriales*, and were inherited by descendants due to incurring an evolutionary advantage. In addition to these trees, we have also constructed a tree based on the sequences of the large subunit from the three main RNR classes (Supplementary Figure 8). The three classes of RNR formed distinct clades in the tree, which were separated from each other by long branches. Based on the midpoint rooting of the tree, the sequences from the class III RNR, which function under strictly anaerobic conditions, were found to form a sister clade to sequences from the classes I and II. The observed branching of the class III RNR in the tree is in agreement with earlier work (Reichard, 1993; Logan et al., 1999; Larsson et al., 2001; Poole et al., 2002; Sintchak et al., 2002; Torrents et al., 2002) suggesting that this class of RNR represents the ancestral form of the reductase. Although phylogenetic analysis can shed light on the evolutionary history of the three types of RNR, it does not explain the variable distribution of these classes in different organisms. As an important protein, there is at least one type of RNR in every organism. However, the combination of different RNRs which are found in various organisms is unpredictable and it does not show any correlation with the evolutionary histories of the organisms (Torrents et al., 2000; Lundin et al., 2009, 2010; Torrents, 2014).

### Locations of the CSIs in the Ribonucleotide Reductase Homologs Structures

**FIGURE 4:**
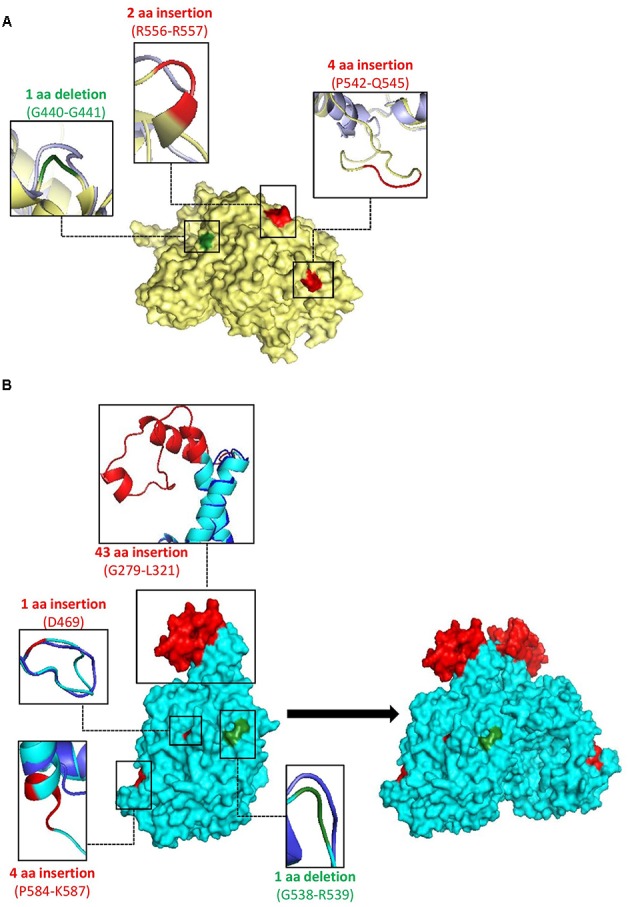
Surface representations of homology models of **(A)** the class Ib ribonucleotide reductase monomer from *Bifidobacterium longum*, modeled from the 1PEQ template (Uppsten et al., 2003); the cartoon representations are of the model (yellow) superimposed on the template (purple) and **(B)** the class III ribonucleotide reductase monomer (NrdD) from *B. longum*, modeled from the 1H7B template (Larsson et al., 2001), with secondary structure in agreement with PSIPRED and CONCORD analyses; the cartoon representations are of the model (cyan) superimposed on the template (dark blue). The observed dimer on the right of **(B)** is a result of docking analysis from ClusPro, refined by ROSIE. The conserved signature insertions identified in these proteins are highlighted in red, and the positions of the deletions are shown in green.

To gain insights into the possible significance of the identified CSIs, homology models for the class Ib and III RNRs from *B. longum* were constructed (see Materials and Methods section) based on previously crystallized template structures of class Ib and III RNR proteins (Larsson et al., 2001; Uppsten et al., 2003). After the validation of the homology models using a variety of tools described under section “Materials and Methods,” a superimposition of the final selected models with the template structures was carried out using PyMOL to determine the locations of the identified CSIs in the structures of the class Ib and III proteins. The locations of the three CSIs identified in class Ib RNR in the modeled structure of the NrdE protein is shown in **Figure 4A**. As seen, all three CSIs in the NrdE homolog were located within the surface loops of the protein (**Figure 4A**). However, of these CSIs, the 2 aa insert also appears to extend a helix. The current model is in agreement with secondary structure analyses (PSIPRED/CONCORD), and yielded reassuring measurements by ERRAT, Verify3D, RAMPAGE and PROSA. The locations of these CSIs in the structure of the *B. longum* NrdE subunit indicate that they are topologically distant from the dimer interface/allosteric regulatory site, as well as the active site as seen in Supplementary Figure 9A (Uppsten et al., 2003, 2006). However, the locations of these CSIs within surface loops in the NrdE structure indicate that they could be involved in mediating novel protein–protein interactions (Cherkasov et al., 2006; Singh and Gupta, 2009; Gupta, 2016; Zhang et al., 2016; Khadka and Gupta, 2017).

Of the five CSIs found in the NrdD homologs (Class III) of bifidobacteria, the structural locations of four indels could be determined and are illustrated on the modeled structure (**Figure 4B**). Structural location of one of the CSIs present near the C-terminal end could not be determined as the structural information for the corresponding region was absent from the template structure (PDB ID: 1H7B) used for homology modeling (Larsson et al., 2001). Similar to the indels found in the NrdE structure, most of the CSIs in the NrdD structure are also be found on surface loops that are structurally distant from the active site (**Figure 4B** and Supplementary Figure 9B). The 4 aa insert, also located on the surface exposed region, appears to form a loop and elongate a helix. The large 43 aa insert in NrdD exists as an elongation of the allosteric regulatory region, in between two helices that form the 4-helix bundle in the NrdD dimer (Uhlin and Eklund, 1994; Logan et al., 1999; Larsson et al., 2001). Since the 43 aa insert did not correspond to a characterized domain or motif, elucidating its structure was a challenging task. We present a model that is in agreement with secondary structure predictions from the *B. longum* primary sequence (PSIPRED/CONCORD) (**Figure 4B**), and an additional model according to the prediction made *in silico* by the I-TASSER server (Supplementary Figure 10B). As seen in Supplementary Figure 10B, in the I-TASSER model, the 43 aa insert appears to form two helices that are connected to one another by a loop, and each are also connected to the two existing helices by loops. The orientation of the insert is such that it folds back toward the bulk of the protein, and the dimer interface is relatively uninfluenced. In the case of the model generated based on the secondary structure predictions, the CSI forms an extension of the two helices, along with two small helices connected by loops in between (**Figure 4B**). In order to be inclusive of all reasonable possibilities, we also modeled the NrdD homolog based on the hypothesis that, instead of small helices with breaks induced by loops according to the PSIPRED/CONCORD model, perhaps the loops connecting the main helices to the small helices may be extended helices without loop-induced breaks (Supplementary Figure 10A). This extended helix hypothesis is a corollary to the observation that the elongated helix in class III compared to class I has important functional significance regarding allosteric binding, and also influences dimer packing (Larsson et al., 2001). All three models were refined and validation scores were maximized in these CSI-containing regions.

### Analyzing the Possible Functional Significance of the Large Conserved Insert

To determine the possible role of the large 43 aa insert in dimer formation or complex stability, we have performed a series of docking studies to reveal dimerization potentials of the models compared to an additional model of bifidobacterial NrdD that lacks the CSI. These four models (I-TASSER, PSIPRED/CONCORD, extended helix hypothesis and CSI-lacking) were submitted to two online servers viz. ClusPro and PatchDock. The complexes obtained from ClusPro and PatchDock were refined and scored using ROSIE, and an additional dimerization measurement was performed by superimposing the models with the available experimentally solved structure of the NrdD dimer (Larsson et al., 2001). The docking scores of the dimer complexes from the protein–protein docking studies are summarized in **Table 1**. The PatchDock server did not yield any dimer complex for the modeled proteins containing extended helix that was plausible with the known biological assembly. However, the docking scores for the CSI-containing protein model based on PSIPRED/CONCORD were consistently improved (or superior) for all servers in comparison to the protein model lacking the CSI. Thus, the model generated according to the PSIPRED/CONCORD prediction is more likely to approximate the true structure of the class III RNR in *B. longum*. For all docking servers, the I-TASSER generated protein model did not form a plausible dimer complex and hence its results are not shown. This may be due to the fact that in the I-TASSER model of NrdD, the CSI was found to protrude away from the dimeric interface (Supplementary Figure 10B).

To examine if any of the residues from the CSI are involved in dimer interaction, the structural coordinate file for the dimeric form of the PSIPRED/CONCORD model was submitted to the PDBePISA server (Krissinel and Henrick, 2007). Analysis of the results obtained suggests that two residues from the large CSI (viz. Gly279, Met280 for *B. longum* NrdD) are present at the protein dimer interface. However, both of these residues, which are present at the N-terminal end of this large CSI, are not conserved in other *Bifidobacteriales* homologs. Thus, it is difficult to infer with any degree of confidence the possible role of these two residues in protein dimerization. Asides from these two residues, the remaining CSI residues are partly or fully solvent accessible near the interface.

## Discussion

The *Bifidobacteriales* are an important group of bacteria that consist of both pathogenic species and health-promoting commensal microorganisms that are frequently exploited in the food industry as probiotics (Gibson et al., 1995; Sanders, 1998; Leahy et al., 2005; Masco et al., 2005; Ventura et al., 2009; Cronin et al., 2011). However, the use of these bacteria as probiotics faces several challenges as very little is understood about the mechanism responsible for the beneficial effects exerted by bifidobacteria (Oberg et al., 2011). In the present work, we have identified several novel signatures in the form of CSIs in the sequences of both classes I and III RNR homologs that differentiate the RNR homologs from *Bifidobacteriales* from all those found in all other organisms. Earlier work on CSIs (including 1–2 amino acid indels) in several important proteins (e.g., GroEL, DnaK, GyrB, PIP5K, etc.) provides evidence that the CSIs play important functional roles in CSI-containing organisms, and deletion or other changes in the CSIs adversely impact cell growth or other critical functions (Chatterji et al., 2000; Singh and Gupta, 2009; Schoeffler et al., 2010; Clarke and Irvine, 2013; Gupta et al., 2017). In this context, the results reported here that both classes I and III RNR homologs harbor multiple CSIs that are uniquely present in all bifidobacteria is of much interest. These CSIs serve to clearly differentiate the bifidobacterial RNR homologs from those found in all other organisms, and they could function in conjunction with each other to impart certain novel functional characteristic(s) that is only shared by the classes I and III RNR homologs from bifidobacteria.

The distribution of RNR homologs in the *Bifidobacteriales* reveals that while all bifidobacteria species contain a class III anaerobic RNR homolog (NrdDG), the class Ib aerobic RNR homologs (NrdEF) were not found (or detected) in a number of *Bifidobacterium* species (viz. *B. adolescentis, B. angulatum, B. dentium*, and *B. gallicum, B. cuniculi, B. lemurum, B. merycicum, B. moukalabense, B. ruminantium*) as well as in members of the genera *Parascardovia* and *Scardovia* (Lundin et al., 2009). The bifidobacteria species lacking the class Ib RNR homologs do not show any specific branching pattern, or belong to any specific clade(s) of bifidobacteria, but they appear to be distributed sporadically within the order *Bifidobacteriales* (**Figure 3**). Thus, it is likely that the genes for both classes I and III RNR were present in the common ancestor of all *Bifidobacteriales* and subsequently some species have lost the genes for the class I RNR. Since the different identified CSIs in the classes I and III RNR homologs are present in all bifidobacteria, the most likely explanation for this fact is that the genetic changes responsible for the observed CSIs occurred in a common ancestor of the order *Bifidobacteriales*, presumably at the time of divergence of this group of bacteria from other organisms, similar to the CSIs in many other proteins that are uniquely found in the members of this order (Zhang et al., 2016).

The biological significance of the wide-spread presence of an aerobic RNR (class Ib) in bifidobacterial species, which are generally regarded as anaerobic organisms, remains to be understood. It is known that bifidobacterial species exhibit varying levels of aerotolerance which affects their viability outside of their natural habitats (e.g., gastrointestinal tract, mouth and vagina of mammals) upon exposure to an oxidative environment (Biavati and Mattarelli, 2006; Oberg et al., 2011). Under these conditions, RNRs are pivotal in maintaining a pool of dNDPs/dNTPs to compensate for DNA and protein damage by reactive oxygen species (ROS), ultimately alleviating the detrimental effects of superoxide stress. Thus, a reasonable assumption is that the class I aerobic RNR may play an important role in the tolerance of bifidobacteria to oxidative environment. Although how bifidobacteria manage oxidative exposure remains to be understood, in few studies that have explored the gene expression of both NrdEFHI and NrdDG systems in *Bifidobacterium* species, including data available from the Gene Expression Omnibus^1^, reveal that, under oxidative stress, the expression of both gene systems was rapidly induced by the altered environment; first, the class Ib system was upregulated, followed by the class III system and other proteins, including proteolytic enzymes (Larsson et al., 2001; Edgar et al., 2002; Xiao et al., 2011; Zuo and Chen, 2016). However, further work is necessary to understand the biological and physiological roles of the class I RNR in bifidobacteria under normal conditions and during oxidative stress.

Earlier work on CSIs in protein structures provides evidence that most of the studied CSIs are located in the surface loops of proteins (Geszvain et al., 2004; Akiva et al., 2008; Singh and Gupta, 2009; Gupta et al., 2017; Khadka and Gupta, 2017). The results of our modeling of the identified CSIs in the NrdE and NrdD protein structures also show that all of described CSIs in the classes I and III RNR protein subunits are present on the surface loops of these proteins (**Figure 4**). Based on extensive earlier work, the surface loops in protein structures often serve as platforms for facilitating novel protein–protein or protein–ligand interactions that are specific for the CSI-containing organisms, without affecting the core functions of the target proteins (Geszvain et al., 2004; Akiva et al., 2008; Singh and Gupta, 2009; Schoeffler et al., 2010; Gupta et al., 2017; Khadka and Gupta, 2017). In a number of cases, the surface loops formed by the CSIs have also been shown to play important role in determining the oligomeric state of the proteins (Itzhaki et al., 2006; Akiva et al., 2008; Hashimoto and Panchenko, 2010). Based on these studies, it is expected that the identified CSIs in the RNR homologs of bifidobacteria will also play novel and functionally important roles that are specific for bifidobacteria. There is no information available at present regarding the biochemical properties of the RNR homologs from bifidobacteria or whether they exhibit any novel functional characteristics. However, the identified CSIs provide highly specific tools for the genetic and biochemical exploration of novel functional characteristics of the RNRs from bifidobacteria.

Of the five different CSIs identified within the NrdD homologs, one of these CSIs is a large 43 aa insert, located proximal to the allosteric regulatory site in the protein monomer (**Figure 4B** and Supplementary Figure 9B) (Larsson et al., 2001). This regulatory site consists of a 4-helix bundle where each monomer contributes two helices. These helices are significantly longer in class III RNRs compared to those of class I (∼21 aa), resulting in an altered dimer packing, mode of effector binding and consequential conformational changes to the active site (Larsson et al., 2001). Due to the presence of the 43 aa CSI in the class III bifidobacterial RNRs in this region, this helical region is further elongated in bifidobacteria, which is suggestive of a feature uniquely shared by these bacteria. In the absence of any information regarding the structure of the NrdD protein from bifidobacteria, or the conformation of this large CSI in the protein structure, it is difficult to predict the precise role that this CSI may play in the RNR structure and/or function. However, the results of our preliminary *in silico* protein–protein docking using three separate docking servers suggest that when the CSI is oriented in such a way that it can interact with the other monomer, the stability of the dimer complex is improved in the presence of the 43 aa CSI, in comparison to the similar docking studies carried out with the protein lacking the CSI (**Figure 4B** and **Table 1**). The results from our *in silico* analyses are broadly suggestive of one possible function of this large CSI. However, a clearer understanding of the functional significances of the identified CSIs should emerge from future biochemical and structural studies on classes I and III RNRs. Nevertheless, the results presented here highlight the many unique sequence features of the bifidobacterial RNRs, whose further investigations could provide important insights into novel functional aspects of these enzymes in bifidobacteria.

## Author Contributions

SA identified some of the conserved inserts, carried out phylogenetic and structural work and wrote the draft manuscript. BK was involved in the structural analysis of the identified conserved inserts and read and commented on the draft manuscript. RG identified the initial CSIs and conceived and directed the entire project and edited the final version of the manuscript.

## Conflict of Interest Statement

The authors declare that the research was conducted in the absence of any commercial or financial relationships that could be construed as a potential conflict of interest. The reviewer PL and handling Editor declared their shared affiliation, and the handling Editor states that the process nevertheless met the standards of a fair and objective review.

## Footnotes

1 http://www.ncbi.nlm.nih.gov/geo/
