# Supplementary material for: Ribonucleotide Reductases from Bifidobacteria Contain Multiple Conserved Indels Distinguishing Them from All Other Organisms: In Silico Analysis of the Possible Role of a 43 aa Bifidobacteria-Specific Insert in the Class III RNR Homolog
Source: Front Microbiol. 2017 Jul 31;8:1409. doi: 10.3389/fmicb.2017.01409 (PMC5535262; doi:10.3389/fmicb.2017.01409)
Supplement: Supplementary file 1 [file Data_Sheet_1.PDF]

			420 457
Bifidobacteriales (>50/>50)	Bifidobacterium bifidum	WP_061871037	HVGRDVSCNLGSLNIAKAMDA GLAGTVETAIRALTSVS
	Alloscardovia criceti	WP_018143396	-----I--------------G ---D---R--Y-----A
	Alloscardovia omnicolens	WP_022857299	-----I-----------T--G --GDS--R--Y--S--A
	Bifidobacterium aesculapii	WP_055426807	--------------------G --------------A-A
	Bifidobacterium animalis	WP_065524024	----------------R---- ----P---------A--
	Bifidobacterium biavatii	WP_051923821	-----I--------------G --G-------------A
	Bifidobacterium bohemicum	WP_033522161	---K-I--------------- ---QP------------
	Bifidobacterium boum	WP_026503141	-----I--------------G ----S----V----A--
	Bifidobacterium breve	WP_065454127	-----I------------I-G ---H------------A
	Bifidobacterium callitrichos	WP_052118989	--------------------G ---D------H---A-A
	Bifidobacterium catenulatum	WP_047750615	--------------------G D-GR---R---------
	Bifidobacterium coryneforme	WP_038459292	-----I--------------G ---D----SV------A
	Bifidobacterium crudilactis	WP_034251492	-----I------------V-N ---D------------A
	Bifidobacterium gallinarum	WP_051917180	Y-------------------- D-GA--R-------A--
	Bifidobacterium kashiwanohense	WP_033501193	--------------------G D-GR---R---------
	Bifidobacterium longum	WP_061870154	-----I-----------T--G --GH------------A
	Bifidobacterium minimum	WP_022861445	--------------------G ---D-----V------A
	Bifidobacterium pseudolongum	WP_022858386	-----I--------------G D--AS-RS------A--
	Bifidobacterium reuteri	WP_044090305	-----I--------------G ---H------------A
	Bifidobacterium saeculare	WP_051916045	YI------------------- D-DA--R-------A--
	Bifidobacterium saguini	WP_033889495	-----I--------------G ---H------------A
	Bifidobacterium subtile	WP_024464121	-----I--------------G --T---S------NA--
	Bifidobacterium thermophilum	WP_044280462	-----I--------------G ---AS----V-------
	Bifidobacterium tsurumiense	WP_026641682	-----I--------------G ----S-S----------
	Gardnerella vaginalis	WP_020759134	-----I--------------G N-GAS-DL-V-------
Other bacteria (0/>500)	Actinobaculum suis	WP_049619637	---K-I-----------T--SPS-GK-----V----A--
	Actinomyces johnsonii	WP_021605879	---K-I-----------T--SPDF-R-IR--V-G--A--
	Actinotignum schaalii	WP_026429159	---K-I--------------SED-GK---I-V----A--
	Agrobacterium rhizogenes	WP_042472844	-M-K-I----------A---SEDFGK-I--------A--
	Arcanobacterium haemolyticum	WP_013169379	---K-I-----------T--SPDFGQ----------A--
	Brucella abortus	WP_032456752	YL-K-I----------A----PDFGK-I--S-----A--
	Cellulomonas cellasea	WP_034634635	-----I------M---L---SPDFGQ----------A--
	Corynebacterium xerosis	WP_046650334	-I-E-I------M---M---SPDFSA-I-----G--A--
	Dietzia alimentaria	WP_010541953	---K-I------M-------SPDFGR-I-----G-SA--
	Gordonia araii	WP_050947274	---K-I------M-------SPD-GQ-I-----G--A-A
	Jonesia quinghaiensis	WP_051196589	EI---I-----------V--GGN-GE--NA------A--
	Labrenzia aggregata	WP_055660872	-L-T-I--------------GGD-GA-----V----A--
	Lawsonella clevelandensis	WP_053962374	-I---I------M-V-M---SPDFGKS---------A--
	Promicromonospora sukumoe	WP_051109624	E----I--------------SPDFGR-ID-------A--
	Sanguibacter keddieii	WP_042437791	-I---I------M-------SPDF-K-ID-------A--
	Tomitella biformata	WP_024795442	E--K-I--------------SPDFGK-IAVS--G--A--
	Bartonella bacilliformis	WP_011807312	T--S-I------M-------SSDFGR-----V----A--
	Celeribacter indicus	AJE45875	RL-T-I--------------GKQ-GQ-----V----A--
	Chromohalobacter japonicus	WP_040241038	RI-E-I-----------T--SSDFGA-------G--A--
	Ewingella americana	WP_034792652	Q--K-I----------S---SPDFGK---M------A--
	Martelella mediterranea	WP_026173633	-M-K-I------M---M---SDDFGR--D-------A--
	Meganema perideroedes	WP_018633359	-L-A-I----------AS--GGN-GA---------SA--
	Mycobacterium ulcerans	OIN18996	K--K-I----------RT--SPDF-Q-I-V------A--
	Nesiotobacter exalbescens	WP_051268911	-L-T-I----------RT--GGD-GA----------A--
	Nitratireductor pacificus	WP_040677418	-L-T-I----------RT--GGD-GA-----V----A--
	Nocardia seriolae	APB00743	K--K-I-----------T--SPD-GK----------A--
	Ochrobactrum intermedium	WP_006472473	YL-K-I----------A---SSDFGK-I--S--S--A--
	Paenirhodobacter enshiensis	WP_036635741	-M-T-I----------AT--GPDFGA-I-A------A--
	Pannonibacter indicus	WP_055454833	-L-K-I-----------T--SDDFGR----S-----A--
	Pelagibaca bermudensis	WP_007797873	RM-T-I-----------S--GRD-GA---A------A--
	Poseidonocella pacifica	SFA79725	EI-A-I-----------T--GRA-GA---------NA--
	Pseudomonas oryzihabitans	WP_059316959	-I-Q-I----------RT--SPD-GR-------G--A--
	Rhodococcus kroppenstedtii	SFA38075	---K-I-----------T--SPDF-Q-I-V---G--A--
	Rhodovulum sulfidophilum	WP_042456362	-L-T-I----------AT--GPDFG----A------A--
	Roseivivax atlanticus	WP_043846938	RM-T-I------M---R---GGD-G-S-T--V---NA--
	Thalassococcus halodurans	SEG49815	-L-T-I-----------T--GPDFGA-----V---SA--
	Vibrio mimicus	WP_042991526	YL-T-I-----------T--GQA-GQS--I-V----A--
	Virgibacillus halodenitrifican	CDQ34016	RI-E-I----------RT--SSDFGA-------G--A--
	Yangia pacifica	SDG07304	KL-T-I-----------T--GPDFGASI--------A--
	Yersinia enterocolitica	KGA78873	-I-K-I----------HI--SPDFGKSI-----G--A--

Supplementary Figure 1. Alignments of a partial sequence from the large subunit of the class Ib ribonucleotide reductase (NrdE) protein containing a single amino acid deletion that is exclusively found in all *Bifidobacteriales* members that carry the homolog, and absent in other bacteria.

Group	Species	Accession	438		480
Bifidobacteriales (>50/>50)	Bifidobacterium bifidum	WP_053824885	GSFKAPMGCRSFLQGWINPETGK	D	EEDGRMNLGVVSVNVPRIA
	Alloscardovia criceti	WP_018142623	-------------N--TD-D--E	A	V--S-L-----T--I----
	Alloscardovia omnicolens	KWZ73401	-------------N---D-D--E	-	V--S-L-----T--I----
	Bifidobacterium adolescentis	CUO26786	-----------------D-K---	-	V----------T--I----
	Bifidobacterium aesculapii	WP_055426883	-----------------------	-	-------------------
	Bifidobacterium angulatum	KFI41264	----------------V------	-	-------------------
	Bifidobacterium animalis	WP_004218231	-----------------D-K---	-	V----------T-------
	Bifidobacterium asteroides	KJY52169	--------------A-T-----E	-	-----------T--I----
	Bifidobacterium biavatii	WP_033495944	-------------------A---	-	-------------------
	Bifidobacterium bohemicum	WP_033521740	----------------V-----E	-	-----------T--I----
	Bifidobacterium bombi	WP_044086850	----------------V------	-	-----------T--I----
	Bifidobacterium boum	WP_026502801	-----------------D-K---	-	V----------T-------
	Bifidobacterium breve	WP_003830182	-----------------------	-	-----------T-------
	Bifidobacterium callitrichos	WP_043165132	-------------------A---	-	-------------------
	Bifidobacterium catenulatum	WP_003834118	-----------------D-A---	-	V----------T-------
	Bifidobacterium choerinum	WP_024541080	-----------------D----E	-	V----------T-------
	Bifidobacterium commune	SCC78037	-------------SS-V-----Q	-	--E--------TL------
	Bifidobacterium coryneforme	AII74262	--------------A-T-----E	-	-----------T--I----
	Bifidobacterium crudilactis	WP_034253261	-----------------D-K--E	-	V----------T--I----
	Bifidobacterium cuniculi	KFI63881	-----------------D----E	-	V----------T--I----
	Bifidobacterium dentium	WP_003839167	-----------------D-K--E	-	V----------T-------
	Bifidobacterium gallicum	WP_006294541	-----------------D----E	-	V----------T-------
	Bifidobacterium gallinarum	WP_033506739	--------------A-TD----E	-	V----------T-------
	Bifidobacterium indicum	WP_033491384	--------------A-T-----E	-	-----------T--I----
	Bifidobacterium kashiwanohense	WP_033500415	-----------------D-A---	-	V----------T-------
	Bifidobacterium longum	WP_010080962	-----------------------	-	-----------T-------
	Bifidobacterium magnum	WP_022859884	-----------------D----E	-	V--S-------T--I----
	Bifidobacterium merycicum	WP_033522224	----------------V------	-	-------------------
	Bifidobacterium minimum	KFI71356	-----------------D-A---	-	V----------T--I----
	Bifidobacterium mongoliense	WP_033512375	-------------------D---	-	-----------T--I----
	Bifidobacterium moukalabense	WP_034874590	-----------------D-K--E	-	V----------T--I----
	Bifidobacterium psychraerophilum	WP_033496056	-----------------D-----	-	V----------T--I----
	Bifidobacterium reuteri	WP_044089753	-----------------------	-	-----------T-------
	Bifidobacterium ruminantium	WP_026646732	----------------TD-K---	-	V----------T--I----
	Bifidobacterium saeculare	WP_033509604	--------------A-TD----E	-	V----------T-------
	Bifidobacterium saguini	WP_033891351	-----------------------	-	-----------T-------
	Bifidobacterium scardovii	WP_033517268	-------------------A---	-	-------------------
	Bifidobacterium subtile	WP_024463669	-------------------K--E	-	-----------T-------
	Bifidobacterium tsurumiense	WP_026642901	----------------KD----E	N	V----------T--I----
	Gardnerella vaginalis	KXI19023	-----------------D-K---	-	V----------T-------
	Parascardovia denticolens	WP_006288597	-----------------D-T--E	-	V--S-L-----T-------
	Scardovia inopinata	WP_006292581	-------------NN-TD----E	-	V--S----------I----
	Scardovia wiggsiae	WP_007147749	-------------N--TD-D--E	-	V--S-L-----T--I----
Other bacteria (27/>500)	Lactobacillus antri	WP_007124653	----T-----------TD-D---	E	VNS--L-----T--L----
	Lactobacillus casei	KTE98198	----------------TD-K---	E	VNA-------IT--L----
	Lactobacillus dextrinicus	WP_057757168	----------------TD----E	E	VNS--------TI-L----
	Lactobacillus harbinensis	WP_027828304	----------------TD-K--E	E	VNA-------LT--L----
	Lactobacillus vaginalis	WP_003717205	----T-----------KD-----	E	VNS--------T--L----
	Cellulosimicrobium funkei	WP_061269386	----V-----------QDEHGQD		VSE--------TL-L----
	Cryobacterium arcticum	ANP74487	----V-----------TDA-GND		VSE--------T--L----
	Demequina aestuarii	WP_062136938	----------------TDEDGND		SV---------TL-L----
	Facklamia ignava	WP_006701278	----------------Y-EAGEE		VNA--------TL------
	Fructobacillus ficulneus	WP_061992995	----T-----------HDE-GKE		VNS--------T--L----
	Granulicatella adiacens	WP_005606955	----V-----------KDENGQE		VN---------TL-I----
	Lactococcus raffinolactis	WP_003138797	----------------QDENGQD		VV---------TL-L----
	Leuconostoc carnosum	WP_014973647	----T-----------QDENGQE		VN------------L----
	Lysinimicrobium flavum	WP_062292461	----------------EDEHGED		SVE--------TL-L----
	Mageeibacillus indolicus	WP_012993756	----V-----------KDENGKE		VNA--------TL-L----
	Oenococcus oeni	WP_002818938	----------------WDESGRE		VNA--------T--L----
	Oerskovia enterophila	KZM35392	----M-----------TDESGED		VVE--------TL-L----
	Pediococcus stilesii	WP_057801693	----------------K-QNGEE		VNS--------TL-L----
	Sporolactobacillus inulinus	WP_010024099	----------------HDENGKE		VNS--------T--L----
	Streptococcus infantarius	WP_006531409	----------------KDENGVE		VNS--------T--L----
	Escherichia albertii	WP_000187764	----T--------GV-E-ENGEQ		IH---N----I-L-L----
	Escherichia coli	KFH77124	----T--------GV-E-ENGEQ		IH---N----I-L-L----
	Photobacterium aphoticum	WP_047874881	-------------GR-TDEN-KE		VHE--N------L-L----
	Photobacterium aquae	WP_047878275	-------------GR-TDEN-KE		VHE--N------L-L----
	Raoultella terrigena	WP_045853314	----T--------GV-E-ED-EQ		VH---N----I-L-L----
	Salmonella enterica	WP_000187810	----T--------GV-E-ENGEQ		IH---N----I-L-L----
	Shigella dysenteriae	WP_000187783	----T--------GV-E-ENGEQ		IH---N----I-L-L----

Supplementary Figure 2. Alignments of a partial sequence from the large subunit of the class III ribonucleotide reductase (NrdD) protein containing a single amino acid insertion that is found in all *Bifidobacteriales* members, and absent in other bacteria. This CSI is also shared by several *Lactobacillus* species, and other Actinobacteria species that are not all presented here.

Group	Species	Accession	530–555
Bifidobacteriales (>50/>50)	Bifidobacterium bifidum	WP_053824885	APTLFRFGAFG RLGASGNVDTLFKNERATVSLGYIG
	Alloscardovia criceti	WP_018142623	-----QH---- --QPGD---Q--NRS---I------
	Alloscardovia omnicolens	KWZ73401	-----QY---- --QPGD---Q--NRS---I------
	Bifidobacterium aesculapii	WP_055426883	------Y---- ----ND-------------------
	Bifidobacterium asteroides	KJY52169	----YQY---- --KPTDS-----R-S----------
	Bifidobacterium biavatii	WP_033495944	----------- ----ND---Q---------------
	Bifidobacterium bohemicum	WP_033521740	------Y---- ----NDS-----R-S----------
	Bifidobacterium bombi	WP_044086850	----Y-Y---- --A-ND---Q--NRS-S--------
	Bifidobacterium callitrichos	WP_043165132	------Y---- ----ND---Q---------------
	Bifidobacterium coryneforme	AII74262	----YQ----- --KPTDS-----R-S-S--------
	Bifidobacterium crudilactis	WP_034253261	-----QY---- --DP-EK--S--RD--S-I------
	Bifidobacterium dentium	WP_065467155	----------- -----DS--Q--R------------
	Bifidobacterium gallinarum	WP_033506739	----------- ----ND---Q---------------
	Bifidobacterium longum	WP_010080962	----------- ----ND---Q---------------
	Bifidobacterium minimum	KFI71356	----------- ----ND-I-Q--R------------
	Bifidobacterium mongoliense	WP_033512375	------Y---- ----NDS--Q--NR--S--------
	Bifidobacterium pseudocatenulatum	WP_034880865	----------- ----NDS--Q--R------------
	Bifidobacterium pullorum	WP_043169019	----------- ----ND---Q---------------
	Bifidobacterium thermacidophilum	WP_029576613	------Y---- ----ND---Q--R------------
	Bifidobacterium thermophilum	WP_044280318	------Y---- ----ND---Q--R------------
	Bifidobacterium tsurumiense	WP_026642901	------H---- ----ND---Q--R------------
	Chlamydia trachomatis	CRH65135	------Y---- --E-NES--Q--R------------
	Gardnerella vaginalis	KXI19023	------Y---- --E-NES--Q--R------------
	Parascardovia denticolens	WP_006288597	-----EH---- --KPTD---V--R-S---I------
	Scardovia inopinata	WP_006292581	-----EH---- --KPTD------NRS-S-I------
	Scardovia wiggsiae	WP_007147749	-----EH---- --KPTD---V-YNRS----------
Coriobacteriales (>20/>20)	Atopobium deltae	WP_066304516	--I-WQY--LA --DKDEPI-K-LYDGYS-I----A-
	Atopobium fossor	WP_028264321	--I-WQY--LA --DKGEPI-K-LYGGYS-I----A-
	Atopobium parvulum	WP_012809209	--I-WQY--LA --DKGEVI-P-LYGGYS-I----A-
	Atopobium rimae	WP_003150363	--I-WQY--LA --DKGEKI-P-LYGGYS-I----A-
	Atopobium vaginae	WP_006302436	--I-WQY--LA --KKGEVI-K-LYDGYS-I----A-
	Collinsella aerofaciens	WP_006234411	--I-WQY--LA --KKGETI-K-LVGGYS-I----A-
	Collinsella intestinalis	WP_006723656	--I-WQY--LA --DKGEKI-K-LFGGYS-I----A-
	Collinsella stercoris	WP_006722099	--I-WQY--LA --DKGETI-K-LYGGYS-I----A-
	Collinsella tanakaei	WP_009140089	--I-WQY--LA --DKGETI-K-LYGGYS-I----A-
	Coriobacterium glomerans	WP_041738569	--I-WQY--LA --SKGETI-R-LFGGYS-I----A-
	Enorma massiliensis	WP_019128027	--I-WQY--LA --DKGETI-K-LYGGYS-I----A-
	Olsenella profusa	WP_021726533	--I-WQY--LA --KKGETI-R-LYGGYS-I----A-
	Olsenella scatoligenes	WP_059053193	--I-WQY--LA --DKGEKI-K-LYGGYS-I----A-
	Olsenella uli	WP_013252350	--I-WQY--LA --RKGETI-R-LYGGYS-I----A-
	Senegalimassilia anaerobia	WP_042436283	--I-WQY--LA --EKGEPI-K-LYGGYS-I----A-
Other bacteria (0/>500)	Lactobacillus ozensis	WP_056966987	--L-YKY----K--NDDDS--E----------V----
	Peptoniphilus lacrimalis	WP_036756270	--I-YQY----R---KEES--Q----R---I------
	Streptococcus anginosus	WP_059221613	--I-YQY----K---KYDK--Q---HR----------
	Aeromonas caviae	KGY67853	--I-YME--C-V--K-DD-ISEI--HG--SI------
	Buttiauxella noackiae	WP_064555416	--I-YME--C-V--K-DDD-SEI---G--SI------
	Cellulosimicrobium cellulans	WP_053369302	--I-YVH----E--APGDD--R--RDG----------
	Commensalibacter intestini	WP_008854996	--I-YTE----V--QTDDEITE----G--SI------
	Cryobacterium arcticum	ANP74487	--I-YVY----QQ-ARTDS------DG-S--------
	Demequina sediminicola	WP_062071011	--I-YVH----R--AVED---D--RDG---I-M----
	Escherichia coli	WP_000187771	--I-YME--C-V--N-DDD-SEI---G--SI------
	Frischella perrara	WP_039103655	--I-YME--C-V--KEDDS-AEI---G--SI------
	Gilliamella apicola	OCG23268	--I-YME--C-V--NEDDS-SEI---G--SI------
	Laribacter hongkongensis	WP_012697728	--I-YME--C-V--K-DDEISDI--HG--SI------
	Listeria monocytogenes	ODB03918	--I-YKY----K--QDGED--Q--NK--S-I-I----
	Lysinimicrobium flavum	WP_062292461	--I-YVH----R---PAEE-----RDG----------
	Mycobacterium abscessus	CPW34583	--I-YMY----K--ATQDA--E----K----------
	Oerskovia enterophila	OCI31938	--I-YVH----E--APDDD--Q--RAG----------
	Pantoea agglomerans	WP_039386885	--I-YME--C-V--N-DDE-GPI-R-G--SL------
	Paraoerskovia marina	WP_052367104	--I-Y-Y----R---PHDA--E--RDG----------
	Photobacterium aphoticum	GAL04200	--I-YTE--L-V--NPDDE-LDI---G--SI-M----
	Providencia rettgeri	KLN45619	--I-YME--C-V--K-DD--AEI---G--SI------
	Sanguibacter suarezii	WP_066460526	--I-YVY----Q--ALNDP-SS---GG----------
	Shewanella amazonensis	WP_011759391	--I-YME--C-V--RPDDDIEPI---G--SI------
	Snodgrassella alvi	WP_037407069	--I-YME--C-V--N-DDS-AQI---G--SI------
	Streptomyces alboniger	KUJ27015	--I-YMH----K--NEDEP--L--NQK-S-I-M----
	Vibrio rotiferianus	WP_038883126	--I-YME--C-V--K-DDSIAEI---G--SI------
	Xylanimicrobium pachnodae	WP_066131615	--I-YKS-G-A --HNGEE-GH--DHG---L---F--

Supplementary Figure 3. Alignments of a partial sequence from the large subunit of the class III ribonucleotide reductase (NrdD) protein containing a single amino acid deletion that is found in all *Bifidobacteriales* members. This CSI is also shared by *Coriobacteriales* species.

Group	Organism	Accession	550		603
Bifidobacteriales (>50/>50)	Bifidobacterium longum	WP_010080962	SLGYIGLAETTAVFYGKNWIRDHGWD	PEGK	EFALSIVKRMNELCKQWSKAEGYH
	Bifidobacterium bifidum	KLN75552	---------A-------D--------	-Q--	----------S-------------
	Alloscardovia criceti	WP_018142623	-------Y--V-M----D-MQ--S--	EQ--	----E--R--HN--VE-DN-----
	Alloscardovia omnicolens	KWZ73401	-------Y--V-M----D-MQ--S--	EN--	K---E--Q--HD--VD-DS-----
	Bifidobacterium adolescentis	CUO26786	-------Y-A-S-----D-M---D--	-Q--	---------------E--D-----
	Bifidobacterium aesculapii	WP_055426883	--------------------------	----	------------------------
	Bifidobacterium angulatum	KFI41264	---------A-------D--------	----	------------------------
	Bifidobacterium animalis	WP_004218231	-------Y-A----F--D-M---A--	----	D------R----------------
	Bifidobacterium asteroides	KJY65678	-------Y-A-S-----D-MK--S--	----	-------R---Q-----E------
	Bifidobacterium biavatii	WP_033495944	--------------------------	----	----------S-------------
	Bifidobacterium bohemicum	WP_033521740	---------A-S-----D--T--S--	----	--------K--A-----E-E----
	Bifidobacterium bombi	WP_044086850	-------Y-A-S-----D-MS--A--	----	-------R---------EN-----
	Bifidobacterium boum	WP_026502801	-------Y-A-------D-M---S--	----	-----------Q-----C------
	Bifidobacterium breve	WP_003830182	--------------------------	----	-------------------S----
	Bifidobacterium callitrichos	WP_043165132	---------A----------------	----	----------S-------------
	Bifidobacterium catenulatum	WP_003834118	-------Y-A-S-------M------	-Q--	---------------E--D-----
	Bifidobacterium choerinum	WP_024541080	-------Y-A-S-------M------	-Q--	----E--R---Q---D--A-----
	Bifidobacterium commune	SCC78037	-------Y-A-S-----D-MT--S--	D---	----------HD--V----E----
	Bifidobacterium coryneforme	AII74262	-------Y-A-S--F--D-MQ--T--	Q---	-------R--SQ-----E-S----
	Bifidobacterium crudilactis	WP_034253261	-------Y-A----F--D-MK--S--	ED--	-------R---Q--A--A-T-R--
	Bifidobacterium cuniculi	KFI63881	-------Y-A---------M------	-Q--	----E--R---D--HE-AA-----
	Bifidobacterium dentium	WP_003841187	-------Y-A-S-------M------	-Q--	-------R-------E-C------
	Bifidobacterium gallicum	WP_006294541	-------Y----M-F--D-M------	-Q--	-------R---------C-E----
	Bifidobacterium gallinarum	WP_033506739	-------Y-A---------M------	----	D-----------------T-----
	Bifidobacterium indicum	WP_033491384	-------Y-A-S--F--D-MQ--T--	Q---	-------R--SQ-----E-S----
	Bifidobacterium kashiwanohense	WP_033500415	-------Y-A-S-------M------	-Q--	---------------E--D-----
	Bifidobacterium magnum	WP_022859884	-------Y-A----F--D-M---D--	ED--	-------RK--L---E-AAD----
	Bifidobacterium merycicum	WP_033522224	---------A-S--------------	----	------------------------
	Bifidobacterium minimum	KFI71356	-------Y-A-S-----D-M------	----	D------R--S---H--A------
	Bifidobacterium mongoliense	WP_033512375	-------Y-A-S--F--H-M------	----	-------R---L------A-----
	Bifidobacterium moukalabense	WP_034874590	-------Y-A-S-------M------	-Q--	-------R-------E-C------
	Bifidobacterium pseudocatenulatum	CUN92267	-------Y-A-S-------M------	-Q--	---------------E--D-----
	Bifidobacterium pseudolongum	WP_026648068	-------Y-A---------M------	-Q--	----E--R---Q---D--A-----
	Bifidobacterium reuteri	WP_044089753	--------------------------	----	------------------------
	Bifidobacterium saeculare	WP_033509604	-------Y-A---------M------	----	D-----------------T-----
	Bifidobacterium saguini	WP_033891351	--------------------------	----	------------------------
	Bifidobacterium subtile	WP_024463669	-------Y-A-S-----D-M------	-Q--	----------TQ-----ENE----
	Bifidobacterium thermophilum	WP_044280318	-------Y-A-------D-M---S--	----	-----------Q-----C------
	Bifidobacterium tsurumiense	WP_026642901	-------Y-A-------D-M------	E---	-----L-R----------A-----
	Gardnerella vaginalis	KXI19023	-------Y-A-S-----D-MQ--S-N	ED--	Q----L-R---Q--Q---------
	Parascardovia denticolens	WP_006291281	-------Y----M----D-MT--S--	E---	----EV-R--HN--AD-E------
	Scardovia inopinata	WP_006292581	-------Y-A--M----D-MT--S--	----	----E--R--HN--V--D-E----
	Scardovia wiggsiae	WP_007147749	-------Y-A--M----D-MT--S--	----	----E--R--HD--VD-DN-----
Other bacteria (0/>500)	Fructobacillus ficulneus	WP_061992995	-------Y--CTA---SH-EDSQEAH		D--EE-----RDY-VE-K-ES---
	Fructobacillus pseudoficulneus	WP_059376311	-------Y--A-A---SH-EQNQEAH		D--EE-----HDY-VE-E-ES---
	Lactobacillus acidipiscis	WP_035631416	-------Y-VGT--F-P--EKNQEAH		D-TV---RSLDAH--R---ET---
	Lactobacillus antri	WP_007124653	-------Y-VCTT---P--EHNQEAH		D--V--T-T-HD--AK-EDE----
	Lactococcus garvieae	BAV02875	-------Y-VATS---PT-ENNKEAK		D-T-E------QD--D---ET---
	Lactococcus lactis	WP_003131195	-------Y-VATT---PT-ENNPEAK		A-TIE-----H-D-ED----S---
	Leuconostoc citreum	WP_004904772	-------Y-VG-Y-F-A--EDNSQAH		D-TVA--R-L--A-Q--E-ED---
	Pediococcus ethanolidurans	WP_057806554	-------Y-VCT----E--EHNAEAH		A--EKV-QQ-RQK-DE-AQ-S---
	Pediococcus parvulus	WP_068807797	-------Y-VCT----E--EHNAQAH		---EKV--A-RQK-DE-AQ-S---
	Sporolactobacillus inulinus	WP_010024099	-------Y-VGT----ST-ESNPEAK		D--I-V--KLW-H-QD-EEEY---
	Streptococcus cristatus	WP_045500228	-------Y-VA-----G--ETNPEAK		D-TIA---D-KRRVEE--EQYD--
	Listeria monocytogenes	ODB31891	-I-----Y-AAT----GE-EG-KEAK		N-T-D---ELKAYADN-KDEY--W
	Paludibacterium yongneupense	WP_036666397	-------H-VGVLMF-QHPFDSPECQ		T-LHE-----SAAT-V--RET-FA
	Pseudogulbenkiania ferrooxidan	WP_021476186	-------H-VGTLLF-SHPADSPNVR		D-LRQV-G---AAAAE-RRET-FG
	Siccibacter colletis	WP_031521685	------VH--INAL---HVYDSEALR		AK--A--E-LRQAVE--KDET--G

Supplementary Figure 4. Alignments of a partial sequence from the large subunit of the class III ribonucleotide reductase (NrdD) protein containing a 4 amino acid insertion that is exclusively found in all *Bifidobacteriales* members, and absent in other bacteria.

Group	Species	Accession	657		713
Bifidobacteriales (>50/>50)	Alloscardovia criceti	WP_018142623	NPKALEAVWDYAHN	I	GIGYLGTNTPIDHC
	Alloscardovia omnicolens	WP_049187680	--------------	-	--------------
	Bifidobacterium actinocoloniiforme	WP_033503616	------------YD	-	--------------
	Bifidobacterium adolescentis	WP_003811121	------------YT	-	------------R-
	Bifidobacterium aesculapii	WP_055426883	------------Y-	V	--------------
	Bifidobacterium angulatum	WP_003827066	------------Y-	-	--------------
	Bifidobacterium animalis	WP_014698108	------------Y-	-	--------------
	Bifidobacterium biavatii	WP_033495944	------------Y-	-	--------------
	Bifidobacterium bifidum	WP_003812105	------------Y-	-	--------------
	Bifidobacterium bombi	WP_044086850	------------Y-	-	--------------
	Bifidobacterium boum	WP_026502801	------------YK	-	--------------
	Bifidobacterium breve	WP_065470960	------------Y-	-	--------------
	Bifidobacterium callitrichos	WP_043165132	------------Y-	V	--------------
	Bifidobacterium catenulatum	WP_003834118	------------Y-	-	------------R-
	Bifidobacterium choerinum	WP_024541080	------------Y-	-	--------------
	Bifidobacterium dentium	WP_003841187	------------Y-	-	--------------
	Bifidobacterium magnum	WP_022859884	------------S-	-	------------R-
	Bifidobacterium merycicum	WP_033522224	------------Y-	-	--------------
	Bifidobacterium mongoliense	WP_033512375	------------Y-	-	--------------
	Bifidobacterium pseudolongum	WP_026643621	------------Y-	-	--------------
	Bifidobacterium reuteri	WP_044089753	------------Y-	-	--------------
	Bifidobacterium ruminantium	WP_034884791	------------Y-	-	------------R-
	Bifidobacterium saeculare	WP_033509604	------------Y-	-	--------------
	Bifidobacterium saguini	WP_033891351	------------Y-	-	--------------
	Bifidobacterium scardovii	WP_033517268	------------Y-	-	--------------
	Bifidobacterium stellenboschense	WP_034530053	------------Y-	V	--------------
	Bifidobacterium subtile	WP_024463669	------------Y-	-	--------------
	Bifidobacterium thermacidophilum	WP_029576613	------------YK	-	--------------
	Bifidobacterium thermophilum	WP_015450017	------------YK	-	--------------
	Bifidobacterium tsurumiense	WP_026642901	------------Y-	-	--------------
	Gardnerella vaginalis	WP_064347456	------------YK	-	--------------
	Parascardovia denticolens	WP_006288597	------T------S	-	--------------
	Scardovia inopinata	WP_006292581	--------------	-	--------------
	Scardovia wiggsiae	WP_007147749	--------------	-	--------------
Other bacteria (2>500)	Xylanimicrobium pachnodae	WP_066131615	----V-------YD	H	E---M-V-V---R-
	Xylanimonas cellulosilytica	WP_050758509	----V-------YD	H	D---M-V-V---R-
	Actinobaculum massiliense	WP_040317119	-L----------YD		RVA---------R-
	Actinobaculum suis	WP_065414586	-L----------YD		RVA---------R-
	Actinomyces neuii	WP_024332399	-T----------YD		KVA---------K-
	Actinotignum schaalii	WP_016441684	-TA---------YD		KVA---------K-
	Allofustis seminis	WP_018658779	------------YD		KV------------
	Alloiococcus otitis	WP_003777597	------------YD		KV------------
	Amphibacillus xylanus	WP_015011029	------------YD		RV------------
	Carnobacterium alterfunditum	WP_034547271	----------F-YD		R-------------
	Cellulomonas carbonis	WP_043602298	------------YD		RV--------V---
	Demequina aestuarii	WP_062136938	--R---------YD		R-----------R-
	Dolosigranulum pigrum	WP_004635282	----------F-YS		RV------------
	Enterococcus asini	WP_010754169	-----------SYD		K-------------
	Facklamia languida	WP_040447792	------------YQ		RV------------
	Fructobacillus tropaeoli	WP_059393624	--Q-------W-YD		HV-------S--K-
	Lacticigenium naphtae	WP_027108380	------------YP		R-A-----------
	Lactobacillus acidifarinae	WP_057801006	----------W-YD		HV-------A--Q-
	Leuconostoc pseudomesenteroides	WP_036086247	--Q------NW-YD		HV-------C--K-
	Listeria floridensis	WP_036095703	-T----------YD		RVA---------K-
	Lysinimicrobium flavum	WP_062292461	------------YD		RV----------R-
	Macrococcus caseolyticus	WP_015912314	-L---------SYD		KV------L---K-
	Melissococcus plutonius	WP_048665585	------T-----YD		KV------------
	Oenococcus alcoholitolerans	KGO32496	----------W-YD		KV-------S----
	Oerskovia enterophila	KZM35392	------------YD		RV----------R-
	Paraoerskovia marina	WP_052367104	--S---------YD		RV----------R-
	Pediococcus parvulus	WP_068807797	----------W-YD		HV-------S--K-
	Trichococcus flocculiformis	CZQ98327	------T---F-YD		KV------------
	Trueperella pyogenes	WP_038103051	-T----------YD		RVA---------K-
	Vagococcus lutrae	WP_023605497	-----------SYD		K-------------
	Varibaculum cambriense	WP_022865485	-T----------YD		KVA---------K-
	Weissella paramesenteroides	WP_002828128	--A-------W-YD		RV------AS----

Supplementary Figure 5. Alignments of a partial sequence from the large subunit of the class III ribonucleotide reductase (NrdD) protein containing a single amino acid insertion that is exclusively found in all *Bifidobacteriales* members, and absent in other bacteria. This CSI is also shared by *Xylanimicrobium pachnodae* and *Xylanimonas cellulosilytica.*

1 MTDLTATGLPLDNTADTAAEQYDPAHDYHALNAMLNLYDADGKIQFDKDKAAEREYVTGH

61 VATNSKRFASAAERLEYLISNQYYNPAVFNQYSAEFLDRIYEHVESAGFEFGTFLGAFKF

Class Ib ribonucleotide reductase

121 YTSYALKTFDGRLYLEDFPQRCAAVALELAAGNEQQAIEYADEMLAGRFQPATPTFLNLG

Class Ib ribonucleotide reductase

181 KAQRGEPVSCFLVRIEDNMESISRGINAALQLSKRGGGVALLLSNLRELGAPIKHIENQS

Class Ib ribonucleotide reductase

241 SGVIPVMKLLEDSFSYANQLGARQGAGAVYLNAHHPDILRFLDTKRENADEKIRIKSLAL

Class Ib ribonucleotide reductase

301 GVVIPDITFELAKQKAQMALFSPYDVERVYGKPFADISVTEHYDEMVADDRIKKTYIDAR

Class Ib ribonucleotide reductase

361 KFFTTIAELQFESGYPYIVFEDTVNRANPIEGRVTMSNLCSEILQVQEPSAYNEDLTYAH

Class Ib ribonucleotide reductase

421 VGRDISCNLGSLNIAKAMD**<u>GG</u>**LGHTVETAIRALTSVAEHTSINAVPSIRRANEEGHAIGL

Class Ib ribonucleotide reductase

481 GQMNLHGFLAREGIQYGSEEGLDFTDMYFMTVAYHAYRASHALAVEHGRTFASFATSDYA

Class Ib ribonucleotide reductase

541 K**<u>PAGQ</u>**GNYFDKYTDG**<u>RR</u>**SLTPRTERVRALFEQYGIAIPTAADWEALRDAILKDGIYNQNL

Class Ib ribonucleotide reductase

601 QAVPPTGSISYINHSTSSIHPIASKIEIRKEGKIGRVYYPAAYMTNDNLGYYKDAYEIGW

Class Ib ribonucleotide reductase

661 KAIVDTYAEATQHVDQGLSLTLFFPDTATTRDLNKAQIYAWRKGIKTLYYIRIRQQALEG

721 TEVQGCVSCML

Supplementary Figure 6. Primary sequence of the NrdE subunit of the class Ib ribonucleotide reductase from *Bifidobacterium longum* (Accession no. EPE39971). The NCBI CD-search server was used to determine the coordinates of the presented domain in purple. The locations of the three identified CSIs are depicted by bold and underlined residues; red indicates an insertion, green indicates the residues surrounding a single amino acid deletion.

ATP cone domain

1 MGAQVLEETVTDNETKVAAKASTVLVEKRDGRVVDFDPINIISAVKSAFGDLNKEVGPEE

ATP cone domain

61 DAMIRGFANQVEGEIKGRYTGPAKIEDIQNLVEHALIDAHLYDVARAYTNYRLDKDIQRA

Class III ribonucleotide reductase

121 KATDVNEAVARFINHDPTLIHENANKDSNVYSTQRDLLAGAVSKAAAFNMLPPAVSNAHM

Class III ribonucleotide reductase

181 KGDIHFHDADYSPFTAQSNCSLPNFWDMLANGFTLGNAPMASPKSIAIAATQITQIMKDV

Class III ribonucleotide reductase

241 ASSQYGGQTANRADEHLAQYAKKDYEKFLEEARETIPD**GMPVEFARRQVESAKKNEPAKL**

Class III ribonucleotide reductase

301 **HFGSREPLPMDTPFHTDVDEL**EQEREILAKIRTRKAIYDAMQTMEYQINSNRVSNGQTPF

Class III ribonucleotide reductase

361 VTVGFGLGTDWFSREVQRAILLNRIRGLGKEHHTAIFPKLVFTVKHGVNADPGDPNYDLK

Class III ribonucleotide reductase

421 QLALESATKRMYPDVVFYENIVKITGSFKAPMGCRSFLQGWINPETGK**D**EEDGRMNLGVV

Class III ribonucleotide reductase

481 TVNVPRIAIESHGDKARFWKLFDERMEVAHQALQFRIMRCKEATPVNAPTLFRFGAF**GR**L

Class III ribonucleotide reductase

541 GANDNVDQLFKNERATVSLGYIGLAETTAVFYGKNWIRDHGWD**PEGK**EFALSIVKRMNEL

Class III ribonucleotide reductase

601 CKQWSKAEGYHYSVYSTPAESLTDRFNRMDREKFGRIEGVTDHDFYTNSFHYPVWLQPTP

Class III ribonucleotide reductase

661 MEKLSYEKDFPYYASGGFINYCEYPCLQDNPKALEAVWDYAYN**I**GIGYLGTNTPIDHCFV

Class III ribonucleotide reductase

721 CGFQGDFEPTEEGFKCPECGNSNPDKCNVTKRTCGYLGSPVQRPMVHGRHEEIAHRVKHM

781 SGETGRVTLDDGTTREWFEETK

Supplementary Figure 7. Primary sequence of the NrdD subunit of the class III ribonucleotide reductase from *Bifidobacterium longum* (Accession no. KXS29127). The NCBI CD-search server was used to determine the coordinates of the presented domains in orange and blue. The locations of the five identified CSIs are depicted by bold and underlined residues; red indicates an insertion, green indicates the residues surrounding a single amino acid deletion.

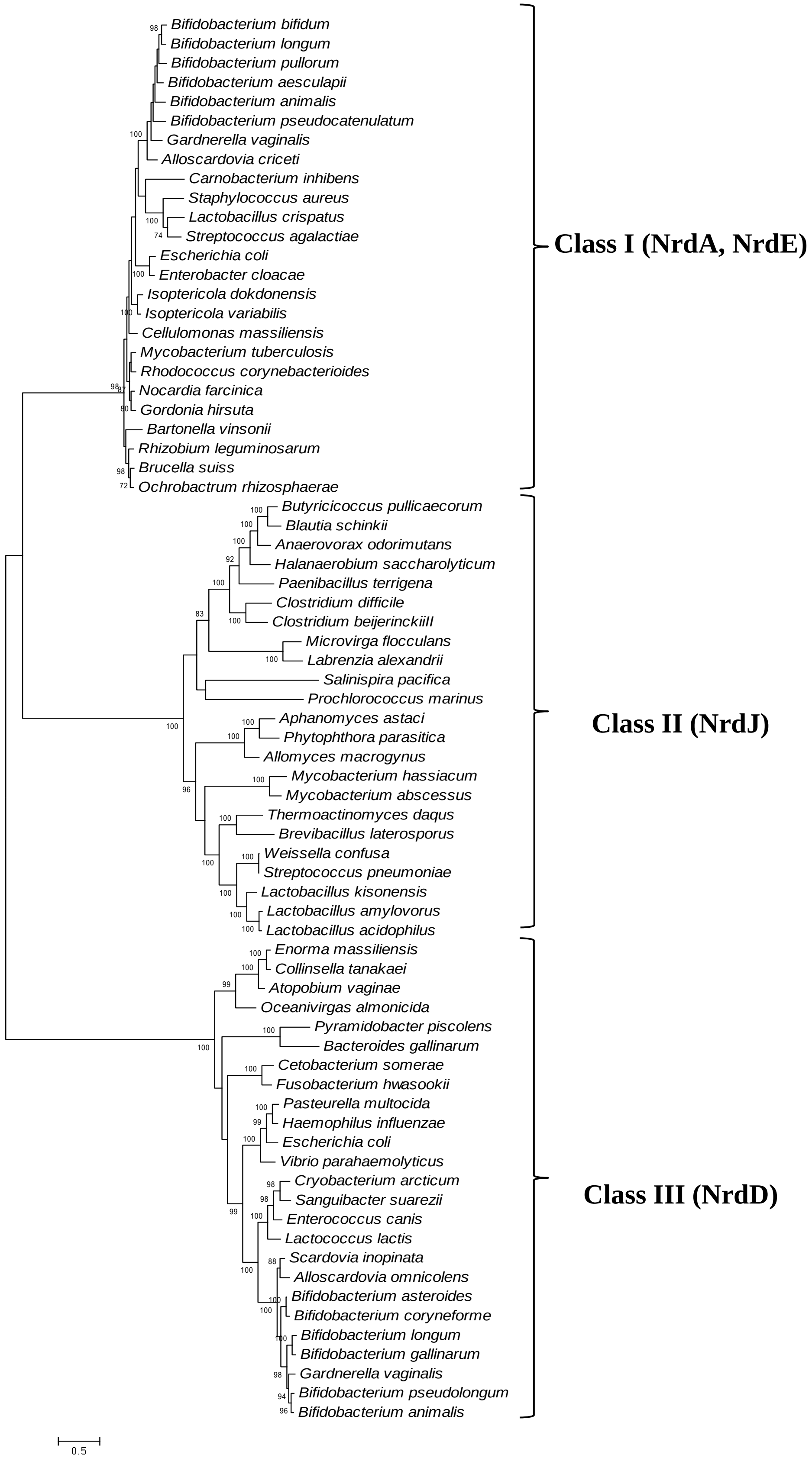

Supplementary Figure 8. A maximum likelihood tree based on sequences of the large subunit of the class I (NrdA and NrdE), class II (NrdJ), and class III (NrdD) ribonucleotide reductases. The tree is based on 324 aa residues after the complete deletion of gaps in the alignment. The tree was constructed using the MEGA6 program and is drawn to scale where horizontal branch length is measured in the number of amino acid substitutions per site. Bootstrap scores that were >70% are indicated as a percentage on the nodes.

(A)

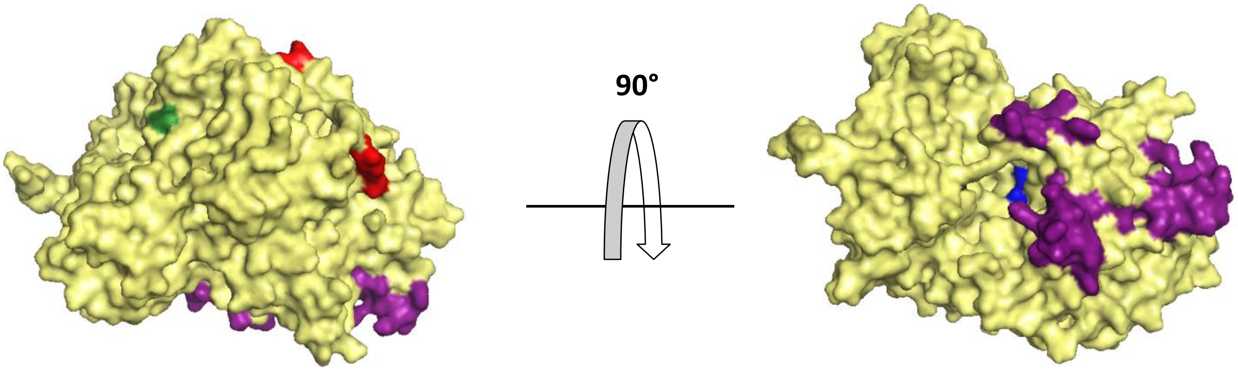

(B)

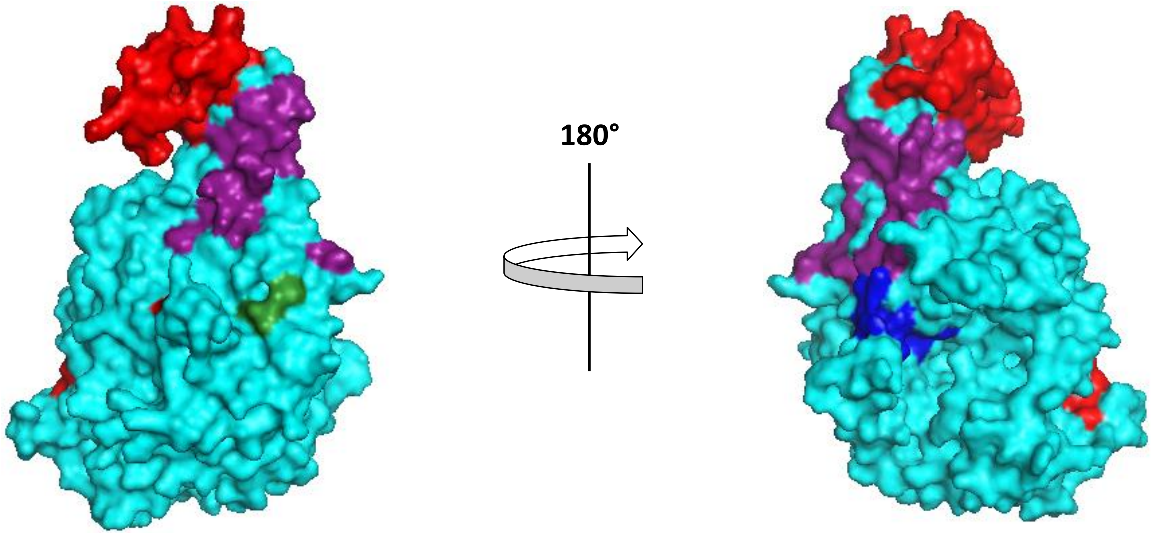

Supplementary Figure 9. Surface representation of (A) the NrdE monomer subunit from the class Ib ribonucleotide reductase of *Bifidobacterium longum* modelled from the 1PEQ template (Uppsten *et al*., 2003) and (B) the NrdD monomer subunit from the class III ribonucleotide reductase from *B. longum*, modelled from the 1H7B template (Larsson *et al*., 2001) according to PSIPRED secondary structure analysis. The conserved signature insertions and deletions identified in the proteins are shown in red and green, respectively. The residues and regions involved in allosteric regulation are highlighted in purple, including the helices that comprise the 4-helix bundle during dimer formation. The active sites are shown in dark blue.

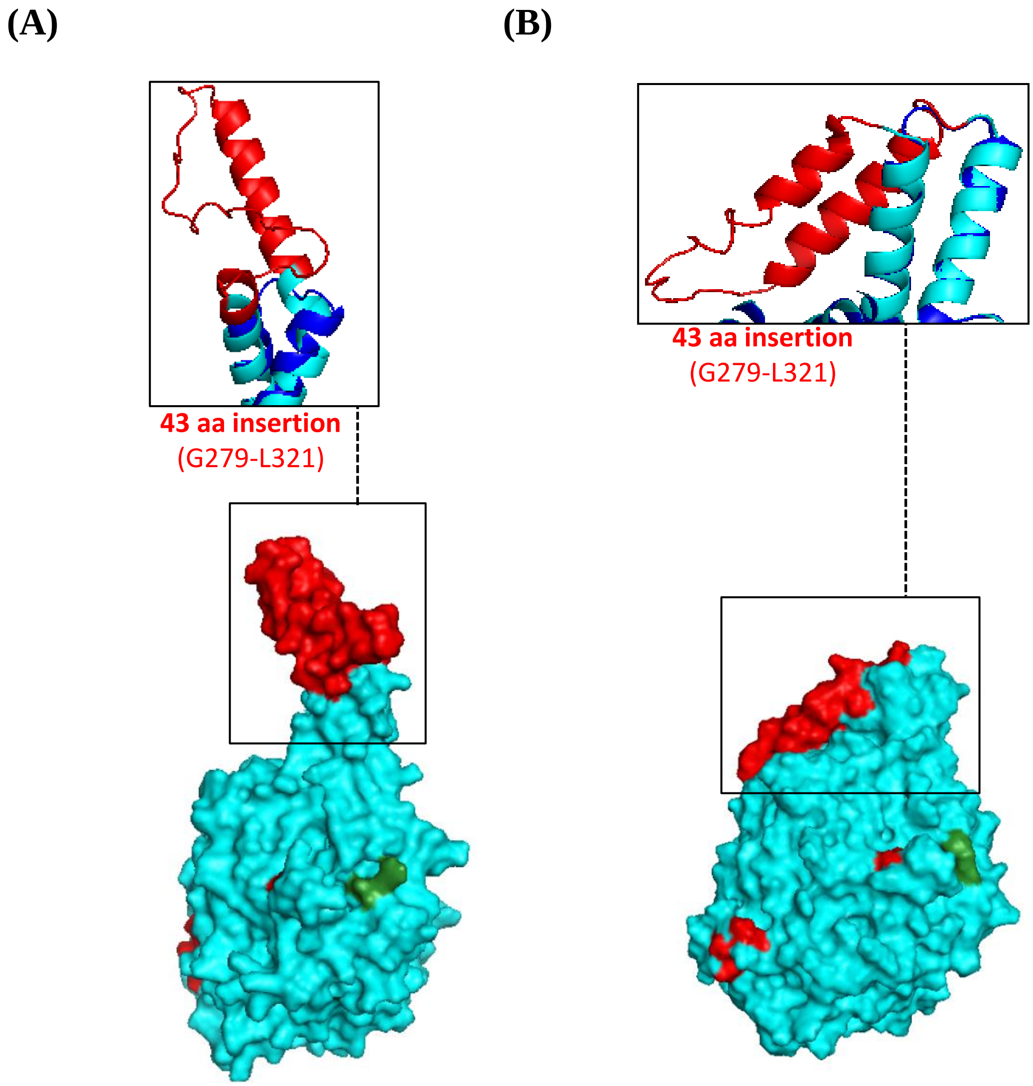

Supplementary Figure 10. Surface representation of the class III ribonucleotide reductase monomer (NrdD) from *Bifidobacterium longum*, modelled from the 1H7B template (Larsson *et al*., 2001), according to (A) the extended helix hypothesis and (B) results from *in silico* modelling by the I-TASSER server. Two of the conserved signature insertions, as well as the deletion, have the same secondary structure as illustrated in Figure 4B. The large insertion differs from the structure in Figure 4B and is illustrated.
